# The role of artichoke leaf tincture (*Cynara scolymus*) in the suppression of DNA damage and atherosclerosis in rats fed an atherogenic diet

**DOI:** 10.1080/13880209.2018.1434549

**Published:** 2018-02-06

**Authors:** Natasa Bogavac-Stanojevic, Jelena Kotur Stevuljevic, Darko Cerne, Janja Zupan, Janja Marc, Zorica Vujic, Milkica Crevar-Sakac, Miron Sopic, Jelena Munjas, Miroslav Radenkovic, Zorana Jelic-Ivanovic

**Affiliations:** a Department of Medical Biochemistry, Faculty of Pharmacy, University of Belgrade, Belgrade, Serbia;; b Department of Clinical Chemistry, Faculty of Pharmacy, University of Ljubljana, Ljubljana, Slovenia;; c Department of Pharmaceutical Chemistry, Faculty of Pharmacy, University of Belgrade, Belgrade, Serbia;; d Department of Pharmacology, Clinical Pharmacology and Toxicology, Faculty of Medicine, University of Belgrade, Belgrade, Serbia

**Keywords:** Oxidative damage, haemeoxygenase-1, monocyte chemoattractant protein-1, gene expression

## Abstract

**Context:** Polyphenols and flavonoids in artichoke leaf tincture (ALT) protect cells against oxidative damage.

**Objectives:** We examined ALT effects on deoxyribonucleic acid (DNA) damage and lipid profiles in rat plasma and gene expression in rat aorta [haemeoxygenase-1 (HO1), haemeoxygenase-2 (HO2), NADPH oxidase 4 (NOX-4), monocyte chemoattractant protein-1 (MCP-1) and nuclear factor (erythroid-derived 2)-like 2 (Nrf2)].

**Materials and methods:** Eighteen male Wistar albino rats were divided into three groups (*n* = 6/group): The control group (CG) was fed with standard pellet chow for 11 weeks; the AD group was fed for a similar period of time with pellet chow supplemented with 2% cholesterol, 3% sunflower oil and 1% sodium cholate. The ADA group was fed with pellet chow (for 1 week), the atherogenic diet (see above) for the following 4 weeks and then with ALT (0.1 mL/kg body weight) and atherogenic diet for 6 weeks. According to HPLC analysis, the isolated main compounds in ALT were chlorogenic acid, caffeic acid, isoquercitrin and rutin.

**Results:** Normalized HO-1 [0.11 (0.04–0.24)] and MCP-1 [0.29 (0.21–0.47)] mRNA levels and DNA scores [12.50 (4.50–36.50)] were significantly lower in the ADA group than in the AD group [0.84 (0.35–2.51)], *p* = 0.021 for HO-1 [0.85 (0.61–3.45)], *p* = 0.047 for MCP-1 and [176.5 (66.50–221.25)], *p* = 0.020 for DNA scores. HO-1 mRNA was lower in the ADA group than in the CG group [0.30 (0.21–0.71), *p* = 0.049].

**Conclusions:** Supplementation with ALT limited the effects of the atherogenic diet through reduced MCP-1 expression, thereby preventing oxidative damage.

## Introduction

Current data inform us that the most common causes of death worldwide are atherosclerosis-associated diseases (Hansson 2005; Go et al. 2014), evidence suggests that common risk factors for atherosclerosis, such as hypercholesterolemia (Jandaghi et al. 2016; Yousefi et al. 2017), hypertension (Ardalani et al. 2016), obesity (Furukawa et al. 2017; Niemann et al. 2017), a sedentary way of life (Gomez-Cabrera et al. 2008) and smoking (Niemann et al. 2017), all increase the production of free reactive oxygen species (ROS) which leads to oxidative modification of deoxyribonucleic acid (DNA), proteins and lipids, causing changes in their regular functions (De Bont and Van Larebeke 2004) which can contribute to further escalation of atherosclerotic processes (Harman 1956). Attenuation of atherosclerosis development requires changes in life-style habits, use of certain drugs and often certain non-pharmacologic supplementation. Artichoke (*Cynara scolymus* L. [Asteraceae]), a thistle-like plant in the aster family, is well known for its antioxidant and plasma lipid-lowering properties (Rondanelli et al. 2013; Sahebkar et al. 2017).

For many years, polyphenols in artichoke leaf were thought to protect against oxidative damage through free radical scavenging (D’Antuono et al. 2015). Polyphenols mainly interact with receptors or enzymes involved in signal transduction events resulting in induction of antioxidant and/or anti-inflammatory effects (Pandey and Ibrahim 2009). Several phytochemicals present in the artichoke leaf regulate the expression of genes involved in cellular defence by the activation of nuclear factor (erythroid-derived 2)-like 2 (Nrf2) (Jeong et al. 2006). Activation of Nrf2 leads to the production of cytoprotective proteins including nicotinamide adenine dinucleotide phosphate (NADPH) dehydrogenase, glutathione *S*-transferase, quinone 1, glutathione peroxidase, glutamate-cysteine ligase, peroxiredoxin I and haemeoxygenase-1 (HO-1) which play crucial roles in cellular defence by facilitating removal of ROS (Mulcahy et al. 1997; Katsuoka et al. 2005; Tosi et al. 2011). In addition, Nrf2 inhibits the expression of pro-inflammatory mediators including cytokines, chemokines and adhesion molecules (Kim et al. 2010). A reduction in ROS and inflammatory mediators prevents oxidative modification of blood lipoproteins and diminishes the risk of arteriosclerosis and coronary heart disease. Furthermore, phenolic acids, including chlorogenic acid, have been shown to affect DNA methylation (Lee and Zhu 2006). Artichoke leaf extract has also been shown to lower total plasma cholesterol, by inhibiting cholesterol biosynthesis in primary cultured rat hepatocytes (Gebhardt 1998). The majority of data regarding the role of polyphenols and flavonoids in artichoke leaf on gene expression in atherosclerosis are from *in vitro* studies. In contrast, the number of studies in animal models is limited.

To further understand the effect of a lipid-rich diet and a lipid-rich diet containing artichoke leaf tincture (ALT) on pathways that contribute to atherosclerotic processes, we investigated the expression of several genes [transcription factor Nrf2, cytoprotective enzymes (HO-1, HO-2), NADPH oxidase 4 (NOX-4) and monocyte chemoattractant protein-1 (MCP-1)] in rat aorta. In addition, we examined stress-induced oxidative DNA damage and key lipid component changes in rat plasma by measuring high-density lipoprotein cholesterol (HDL-cholesterol) and triglyceride (TG) concentrations and calculating the atherogenic index, log (TG/HDL-cholesterol) ratio, a known marker for the presence of small dense low-density lipoprotein particles (sdLDL).

## Materials and methods

For experimental purposes, we used commercial ALT produced by the Institute for Medicinal Plant Research “Dr. Josif Pančić”, Belgrade, Serbia. ALT was prepared from dried primary rosettes from artichoke plants. Single percolation extraction during 24 h was performed using a mixture of ethanol and water (38:62, w/w). The extract was left for 3 d and then filtered. The ALT contained 20% of the plant material by weight. According to the manufacturer for medicinal purposes, the recommended dose of ALT is 30 drops of tincture three times per day or 90 drops daily. For a grown up adult with an average body weight of 70 kg, the dose of ALT is 1.29 ALT drops per kg body weight. Artichoke tincture which we used contained 20 drops/mL, so the exact calculation in mL for 1.29 drops was 64.5 µL/kg per body weight. For ease of measurement and possible loss during treatment, the dose of ALT for rats was round to 100 µL/kg per body weight. The dose was calculated using the rats’ average body weight. Doses of ALT were recalculated once a week, according to body weights of the rats.

Eighteen male Wistar albino rats (2 months old, weighing 150–190 g at the beginning of the experiment) were obtained from the Military Medical Academy Farm (Belgrade, Serbia). They were housed in groups of three in a controlled environment with 12 h light/dark cycles and were allowed free access to food and water. They were randomly divided into three groups (*n* = 6/group). Group 1, the control group of rats which received standard pellet chow (CG) for 11 weeks; group 2, rats fed with normal pellet chow for 1 week and changed to an atherogenic diet (standard pellet chow supplemented with 2% cholesterol, 3% sunflower oil and 1% sodium cholate) for the next 10 weeks (AD); group 3, rats fed with a normal diet for 1 week, the atherogenic diet for the next 4 weeks and then fed with ALT (0.1 mL/kg body weight for 6 weeks (ADA) in the continued presence of the atherogenic diet. ALT extract analysis showed that the content of both cynarin and chlorogenic acid was 0.2%.

All experimental procedures and protocols conformed to institutional guidelines for the care and use of animals in research (Ethics Committee of the Faculty of Pharmacy, University of Belgrade and license number 5/10).

At the end of the feeding period, rats from each group were fasted overnight and sacrificed the following morning. Blood was immediately collected from the heart into heparinized tubes. Whole blood was centrifuged for 10 min at 3000 rpm; plasma was separated, frozen and stored at −80 °C until analysis. Portions of aorta were excised and immediately frozen in liquid nitrogen and kept at −80 °C until RNA isolation was performed.

In plasma, both TG and HDL-cholesterol were assayed by routine enzymatic methods using an ILab 300 + analyser (Instrumentation Laboratory, Milan, Italy) and Randox Laboratories (Armdore, UK) reagents.

The quality of isolated aorta RNA was determined by measuring absorbance ratios at 260 and 280 nm (A260/280) as well as 260 and 230 nm (A260/230) to estimate protein and organic impurities, respectively. Samples with an A260/280 ratio greater than 1.8 and an A260/230 ratio greater than 2.4 were used. RNA integrity was evaluated by electrophoresis. Samples with both subunits clearly visible were used. All extracted RNAs were then stored at −80 °C prior to reverse transcription (RT) reactions.

RT and real-time PCR were carried out on the 7500 Real-Time PCR System (Applied Biosystems, Foster City, CA) using TaqMan^®^ reagent-based chemistry. RT was performed with MultiScribe™ Reverse Transcriptase, random primers and RNase inhibitor using a high-capacity cDNA reverse transcription kit (Applied Biosystems, Foster City, CA) following the instructions from the manufacturer.

Quantitative real-time PCR was performed using TaqMan^®^ 5′-nuclease gene expression assays (Applied Biosystems, Foster City, CA) for HO1, HO2, NOX-4, MCP-1 and Nrf2 genes. Nine reference genes were examined (GADPH, ACTB, TBP, SFN, B2M, HPRT1, UCB, HMBS and PPIA). The geNorm VBA applet for Microsoft Excel was used to determine the most stable gene from the set of tested genes. HMBS was proved to be the most stable housekeeping gene for all three tissues. The results were analyzed in real-time mode using SDS Version 1.4.0.25 software (SPSS, Chicago, IL). Data were expressed as a ratio between the target gene mRNA level and the mRNA level of the housekeeping gene.

Part of the fresh whole blood was used for the determination of single- and double-strand breaks in DNA using the single-cell gel electrophoresis (comet assay). The alkaline comet assay was performed as originally described by Singh et al. (1988) with slight modifications (Sopić et al. 2014). Whole blood was mixed with 0.67% w/v low melting point agarose (Serva, Reno, NV) and placed onto a slide (76 × 26 mm) previously coated with 1% w/v normal melting agarose (Serva, Reno, NV) and covered with a coverslip (24 × 50 mm). After cooling to 4 °C, the coverslip was removed and a layer of 0.5% w/v low melting point agarose was added and left at 4 °C for 5 min. Slides were then dipped into precooled lysis solution [2.5 M NaCl (Serva, Reno, NV), 100 mM Na_2_EDTA (Merck), 10 mM Tris (Merck, Kenilworth, NJ), 1% Triton X-100 (Serva, Reno, NV), pH adjusted to 10 with NaOH (Serva, Reno, NV)] and left overnight in the lysis solution at 4 °C for removal of cellular proteins. After lysis, slides were placed in a submarine electrophoresis tank with fresh alkaline electrophoresis buffer (1 mM Na_2_EDTA, 3 mM NaOH, pH 13.5) for 30 min at 4 °C which allows the DNA to unwind and express alkali-labile damage. Electrophoresis was conducted at 4 °C for 30 min, 25 V and 300 mA, using an Amersham-Pharmacia biotech electrophoresis power supply – EPC 601. Slides were washed with a neutralizing solution [0.4 M Tris, pH 7.5, adjusted with HCl (Serva, Reno, NV)] and then placed in methanol for 30 min for slide preservation. Preserved slides were kept at 4 °C (Woods et al. 1999). Before examination the slides were regenerated in cold water for 30 min, dried out, and stained with ethidium bromide (Serva, Reno, NV). Slides were examined using an Olympus BX 50 microscope (Olympus, Tokyo, Japan). For every sample, two slides were prepared. On every slide 50 comets were scored (100 comets per sample). The cells were classified [according to the method suggested by Collins et al. (1997)] into five classes 0–4 (from undamaged = 0, to maximum damaged = 4). The visual score of each class was calculated by multiplication of the percentage of cells in the appropriate comet class by value of the class. The total visual comet score (DNA score) was the sum of the scores in the five comet classes. Thus, DNA score range from 0 (all undamaged cells) to 400 (all maximally damaged cells). Every slide was scored independently by two people. The average score for every sample was calculated to minimize subjectivity of obtained results. Prior to this study, we performed a dummy run to examine variance within our method. The intra-assay and inter assay coefficients of variance were 4.6% and 8.5%, respectively.

The content of cynarin and chlorogenic acid in ALT was determined by a high-performance liquid chromatography (HPLC). The HPLC system consisted of an Agilent Technologies HP 1200 (binary pump) and a DAD detector (Agilent, Santa Clara, CA). For chromatographic separation a ZORBAX Eclipse Plus C18 Analytical (4.6 × 250 mm, 5 μm) (Agilent, Santa Clara, CA) column was used. A gradient was generated using two solutions: A [phosphoric acid R: water R (0.5: 99.5, v/v)] and B [phosphoric acid R: acetonitrile R (0.5: 99.5, v/v)] (1st min −92% mobile phase A and 8% mobile phase B; 1–20th min linear change from 92% to 75% mobile phase A and from 8% to 25% mobile phase B; 20–33rd min 75% mobile phase A and 25% mobile phase B; 33–35th min linear change from 75% to 0% mobile phase A and from 25% to 100% mobile phase B). The flow rate was 1.2 mL/min. The column temperature was set at 40 °C and the detection wavelength was 330 nm. Reference solutions of cynarin and chlorogenic acid were prepared in methanol (Serva, Reno, NV) and the concentration of both compounds was 0.025 mg/mL. ALT for HPLC analysis was prepared by diluting 1 mL of ALT to 10 mL with a mixture of water and methanol (50:50, v/v). The prepared solution (1 mL) was further diluted to 5 mL with the same diluent.

## Statistical methods

Comparisons of variables were performed using the Kruskal–Wallis test with Mann–Whitney *U* test for subgroup differences. The characteristics of the study populations are presented as median values and interquartile range. Due to the dataset being small, necessary assumption for the asymptotic method was not met. Therefore, we used Monte Carlo simulation. The exact significance level in the Monte Carlo method was calculated by repeatedly sampling from a reference dataset (Roff and Bentzen 1989). Spearman correlation analysis was conducted to determine relationships between examined parameters. Statistical analyses were performed using PASW^®^ Statistic version 22 (SPSS Inc., Chicago, IL) and Microsoft^®^ Office Excel 2007. A value of *p* < 0.05 was considered statistically significant.

## Results

**Figure 1.**
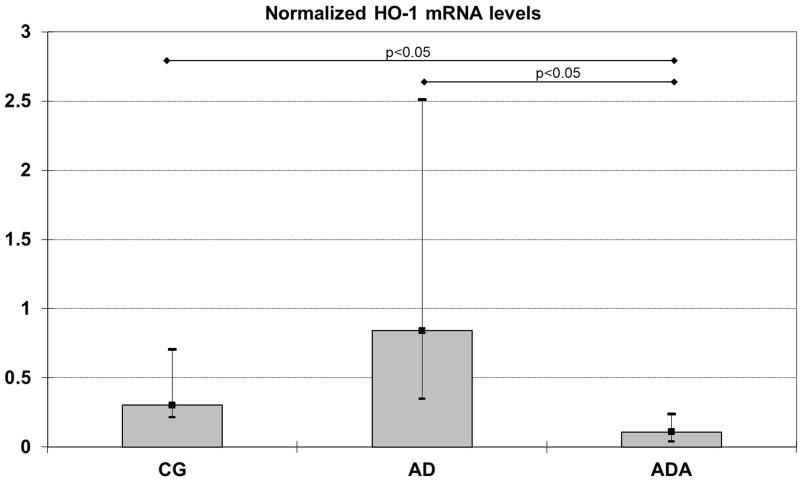
Normalized HO-1 mRNA levels in aortas from rats fed different diets (after 10,000 repeated samples in Monte Carlo simulation). *Significantly lower expression of HO-1 than in CG (*p* = 0.049). **#**Significantly lower expression of HO-1 than in AD (*p* = 0.021).

**Figure 2.**
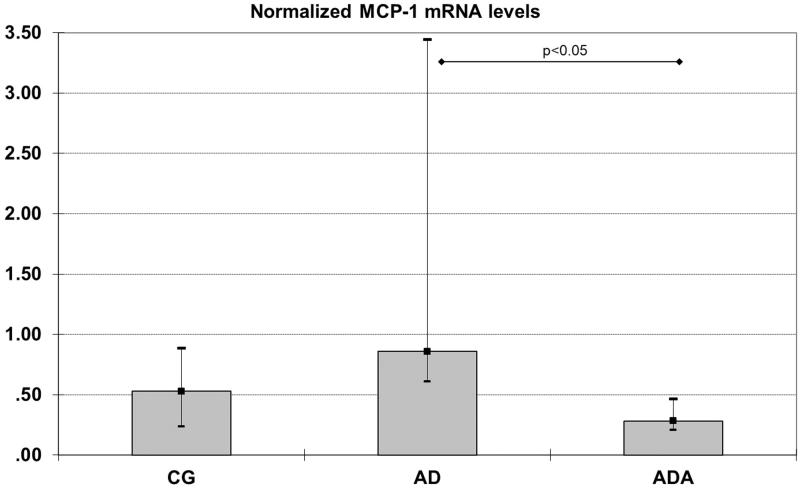
Normalized MCP-1 mRNA levels in aortas from rats fed different diets (after 10,000 repeated samples in Monte Carlo simulation). *Significantly lower expression of MCP-1 than in AD (*p* = 0.047).

**Figure 3.**
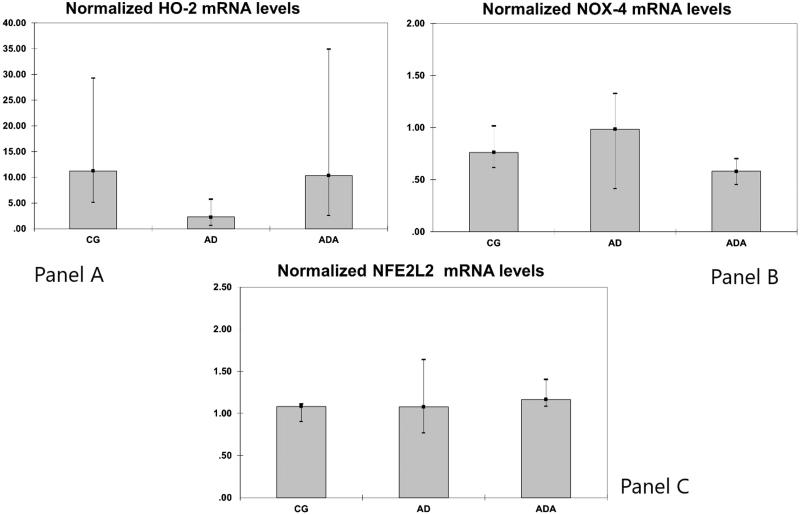
Normalized HO-2 mRNA (panel A), NOX-4 mRNA (panel B) and NLF2L2 mRNA (panel C) levels in aortas from rats fed different diets (after 10,000 repeated samples in Monte Carlo simulation).

Gene expression of HO1, HO2, NOX-4, MCP-1 and Nrf2 were measured in rat aortas. Levels of both HO1 and MCP-1 mRNA were significantly different between groups (*p* = 0.014 and *p* = 0.049, respectively). The normalized level of HO-1 mRNA in the ADA group [0.11 (0.04–0.24)] was significantly lower than the level in both the CG [0.30 (0.21–0.71), *p* = 0.049] and AD [0.84 (0.35–2.51), *p* = 0.021] groups (Figure 1). The normalized MCP-1 mRNA level was higher in the AD group [0.85 (0.61–3.45)] than in the ADA group [0.29 (0.21–0.47), *p* = 0.047] (Figure 2). There were no significant differences in the mRNA levels of HO2, NOX-4 and Nrf2 between the three groups (Figure 3).

**Figure 4.**
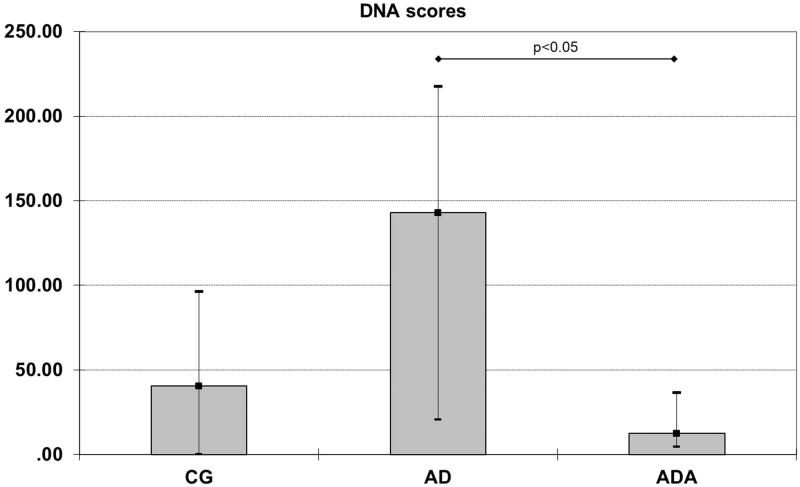
DNA scores in aortas from rats fed different diets (after 10,000 repeated samples in Monte Carlo simulation). *Significantly lower DNA score than in AD (*p* = 0.020).

In order to assess DNA damage caused by reactive oxygen species generated upon the atherogenic diet DNA scores were measured. DNA scores were different according to the type of diet (*p* = 0.036). Rats fed an atherogenic diet had increased DNA scores [176.5 (66.50–221.25)] compared with the atherogenic diet supplemented with ALT [12.50 (4.50–36.50), *p* = 0.020] (Figure 3). There was no difference in DNA score between the CD and AD groups (Figure 4).

Plasma lipid parameters are shown in Table 1. HDL-cholesterol levels across the CG, AD and ADA groups were comparable, while the TG level was significantly lower in the ADA group compared to the CG group (*p* = 0.023). In addition, the atherogenic log (TG/HDL-cholesterol) ratio was significantly lower in the ADA group compared to the CG group (*p* = 0.014).

**Table 1.** Lipid status of Wistar rats on different diet regimes.

Parameter	CG	AD	ADA	*p*
HDL-cholesterol, mmol/L	0.49 (0.41–0.53)	0.42 (0.36–0.58)	0.45 (0.34–0.55)	0.724
TG, mmol/L	0.79 (0.57–0.89)	0.52 (0.44–0.65)	0.39 (0.32–0.56)^a^^,^*	0.042
log(TG/HDL-cholesterol)	0.21 (0.034–0.290)	0.076 (−0.017 to 0.180)	−0.014 (−0.088 to 0.112)^a^^,^*	0.018

All results are presented as median and interquartile range.

a Significantly lower than CG.

* *p* < 0.05. Kruskall–Wallis test than Mann–Whitney *U* test. After 10,000 repeated samples in Monte Carlo simulation.

Associations between lipid parameters, DNA scores and gene expression of HO1, HO2, NOX-4, MCP-1 and Nrf2 were tested using Spearman correlation analysis. In the CG group, DNA scores highly negatively correlated with Nrf2 (*r* = −0.812, *p* = 0.049) gene expression. In the AD group HDL-cholesterol positively correlated with NOX-4 (*r* = 0.940, *p* = 0.048) and Nrf2 (*r* = 0.894, *p* = 0.041) gene expression. However, HDL-cholesterol negatively correlated with HO-1 (*r* = −0.949, *p* = 0.048) gene expression.

HPLC analysis of the ALT revealed the concentrations of some of the compounds present in the ALT. Phenolic acids: chlorogenic acid (778.7 μg/g) and caffeic acid (43.8 μg/g) and flavonoids: isoquercitrin (1388.2 μg/g) and rutin (309.9 μg/g). Gallic acid, hyperoside, quercetin, sakuranetin and rosmarinic acid were not detected in the extract.

## Discussion

Recent studies have expanded our knowledge regarding the complex molecular mechanisms involved in atherogenesis. Dyslipidemia is one of the well-established atherosclerotic risk factors found in a Western high-fat diet (Leon and Bronas 2009). There have been numerous attempts to implement lipid-lowering and antioxidative supplementation in diets in order to suppress atherosclerosis development (Varady and Jones 2005). However, convincing effects of food-derived supplements and their mechanisms of antioxidant or anti-inflammatory behaviour are far from complete and accepted.

Our current study has demonstrated positive effects of ALT on both the plasma lipid profile and oxidative stress status beneficial for the arterial wall. ALT’s phenolic acids (clorogenic and caffeic acid) and flavonoids (isoquercitrin and rutin) are known to have antioxidant properties. The DNA single and double strand break score was lowest in the ADA group, indicating that ALT-derived antioxidants managed to attenuate the harmful effects of the atherogenic diet. In agreement with our findings, a moderate but significant upregulation of DNA repair capacity in lymphocytes was found after dietary supplementation with plants rich in phenolic acids and flavonoids (Guarrera et al. 2007). Despite the abundance of biological data demonstrating the antioxidant activities of chlorogenic acids, controversy persists around the notion that these compounds may be a potent antioxidants or pro-oxidants – depending on their concentration (Morishita and Ohnishi 2001). Zan et al. (2013) demonstrated that high concentrations of leaf and flower extracts of *C. scolymus* L. induced DNA damage in blood cells. These results indicate that artichoke tea should be consumed moderately. Similarly, flavonoids have been reported to exert pro-oxidant activity by elevation of free radicals (Cao et al. 1997).

On the other hand, there is evidence that the antioxidant function of ALT may be mediated via the antioxidant responsive element (ARE/EpRE) by activation of the transcription factor Nrf2 (Jeong et al. 2006). Increased ROS can affect gene expression via translocation of Nrf2 to the nucleus where it can cause upregulation of certain antioxidant genes for example HO-1 (Araujo et al. 2012). Although the AD group showed greater DNA damage than the other groups, there was no increase in the Nrf2 mRNA level. Only in the CG group was there negative correlation of Nrf2 mRNA gene expression and DNA score. Therefore, an atherogenic diet means increased oxidative stress/DNA damage without change in Nrf2 mRNA gene expression, but with increased HO-1 mRNA expression. Associations between HO-1 gene expression and hydrogen peroxide have been documented in other papers (Applegate et al. 1991). ALT supplementation did not influence the level of Nrf2 mRNA. In our recent paper (Crevar-Sakac et al. 2016), we showed that ALT possesses antioxidative effects and that effects were probably sufficient enough to decrease HO-1 and MCP-1 mRNA expression. HO-1 expression is regulated at the transcriptional level and the regulation of HO-1 mRNA in response to oxidative stress may be induced by both transcriptional and post-transcriptional modulations that act independently (Gozzelino et al. 2010; Keene 2010).

MCP-1 is a key monocyte chemokine in atherosclerosis (Gu et al. 1998). Using MCP-1- or MCP-1 receptor-deficient mice to examine atherosclerosis, it was demonstrated that, in the absence of MCP-1 or its receptor, there was a substantial reduction in arterial lipid deposition (Boring et al. 1998). MCP-1 is induced in response to the activation of inflammatory NF-κB pathways in macrophages. As flavonoids possess various anti-inflammatory properties, they are potent inhibitors of NF-κB signalling (Rahman et al. 2006), but are also potent activators of Nrf2 mRNA. Our data are consistent with the idea that ALT suppresses inflammation and ROS production and particularly MCP-1 expression. It was also found that HO-1 attenuated MCP-1 mRNA expression in U937 human monocytic cells (Shokawa et al. 2006). MCP-1 mRNA, which can be induced by oxidative stress (Reape and Groot 1999), was not altered by the atherogenic diet. Therefore, we can presume that upregulation of HO-1 attenuated MCP-1 mRNA expression (Shokawa et al. 2006) and in doing so weakened HO-1’s capability to compensate stress induced by the atherogenic diet. There was no increase in gene expression of pro-atherogenic MCP-1 and there was no increase in HO-1 mRNA expression, suggesting a protective role of ALT.

According to previous studies, certain saturated fatty acids stimulate adipocytes to generate ROS in a NOX-4-dependent fashion linking NFκB activation and expression of monocyte chemotactic factor genes (Yeop et al. 2010). In a study by Umemoto et al. (2013), pre-exposure of cells to HDL inhibited the translocation of NOX-4 to lipid deposits in the plasma membrane and decreased ROS generation. None of the treatments in our study induced any changes in NOX-4 mRNA or HDL-cholesterol. However, in the AD group, *NOX-4* gene expression was positively associated with HDL-cholesterol. Further studies will lead to a deeper understanding of its role in atherosclerosis.

A high logTG/HDL-cholesterol ratio (i.e., high atherogenic index) shows tendency towards sdLDL particle formation which is more atherogenic than large, buoyant particles (Dobiasova and Frohlich 2001). In our current study, we found a significantly lower logTG/HDL-cholesterol ratio in the ADA group compared with the CG group. In addition, ALT caused a significant decrease in TG. According to a study by Shimoda et al. (2003), artichoke extract inhibits gastric emptying which could in part explain its hypotriglyceridemic influence. A study by Qiang et al. (2012) indicated that ALE could decrease both TG and cholesterol in hamsters only after prolonged atherogenic feeding. Several other studies which analyzed compound rich in flavonoids and antioxidants came to similar results. Namely, a study from Jandaghi et al. (2016) demonstrated that *Melissa officinalis* supplementation as a rich source of antioxidants and bioactive compounds can be effective in remission of LDL and AST levels in patients with borderline hyperlipidemia, while Yousefi et al. (2017) showed that Fenugreek seeds supplementation, as a phenolic-rich herb, can be effective in the reduction of some lipid profile in patients with borderline hyperlipidemia. Furthermore, antihypertensive activity of *Rhus coriaria* fruits could be attributed to flavonoids, which are the main chemical constituents of this plant, demonstrating the powerful cardiovascular protective activity of these compounds (Ardalani et al. 2016).

The qualitative and quantitative variability of phenolic and flavonoid content in ALT depends on plant genetic diversity, the physiological stage of plant development (harvest time), climatic conditions during plant growth, extract production (Ceccarelli et al. 2010) and applied dose (Jacociunas et al. 2012). We used the commercial ALT dosage to imitate conditions recommended for human use in the case of dyslipidemia treatment. However, additional studies are needed to explore and compare the influence of different ALT doses on molecular pathways that contribute to atherosclerotic processes.

## Conclusions

ALT exerts its beneficial effect on the arterial vessel wall through a reduction in oxidative stress and by establishing a more favourable plasma lipid profile. ALT’s effect on an atherogenic diet appears to be mediated through diminished MCP-1 expression (DNA damage reduction) and a lowered atherogenic TG/HDL-cholesterol ratio. The atherogenic diet induced HO-1 mRNA expression most likely in response to increased oxidative stress. The antioxidative effects of ALT were clearly sufficient to inhibit the atherogenic diet-induced increase in HO-1 mRNA. This *in vivo* study confirms the results from previous *in vitro* studies. It would be interesting to perform a similar study in humans using their peripheral blood mononuclear cells as distinct vessel-wall imprints.
